# Novel Insights into Inkjet Printed Silver Nanowires Flexible Transparent Conductive Films

**DOI:** 10.3390/ijms22147719

**Published:** 2021-07-19

**Authors:** Yuehui Wang, Xiaoli Wu, Ke Wang, Kaiwen Lin, Hui Xie, Xiaobing Zhang, Jingze Li

**Affiliations:** 1 Department of Materials and Food, Zhongshan Institute, University of Electronic Science and Technology of China, Zhongshan 528402, China; 201921030315@std.uestc.edu.cn (X.W.); wangke1978@zsc.edu.cn (K.W.); 201610102331@mail.scut.edu.cn (H.X.); zhangxiaobin@redsolar.com.cn (X.Z.); 2 School of Materials and Energy, University of Electronic Science and Technology of China, Chengdu 610054, China; lijingze@uestc.edu.cn

**Keywords:** silver nanowires ink, flexible transparent conductive films, inkjet printing, well-defined pattern

## Abstract

Silver nanowire (AgNWs) inks for inkjet printing were prepared and the effects of the solvent system, wetting agent, AgNWs suspension on the viscosity, surface tension, contact angle between ink droplet and poly(ethylene) terephthalate (PET) surface, and pH value of AgNWs ink were discussed. Further, AgNWs flexible transparent conductive films were fabricated by using inkjet printing process on the PET substrate, and the effects of the number printing layer, heat treatment temperature, drop frequency, and number of nozzle on the microstructures and photoelectric properties of AgNWs films were investigated in detail. The experimental results demonstrated that the 14-layer AgNWs printed film heated at 60 °C and 70 °C had an average sheet resistance of 13 Ω∙sq^−1^ and 23 Ω∙sq^−1^ and average transparency of 81.9% and 83.1%, respectively, and displayed good photoelectric performance when the inkjet printing parameters were set to the voltage of 20 V, number of nozzles of 16, drop frequency of 7000 Hz, droplet spacing of 15 μm, PET substrate temperatures of 40 °C and nozzles of 35 °C during printing, and heat treatment at 60 °C for 20 min. The accumulation and overflow of AgNWs at the edges of the linear pattern were observed, which resulted in a decrease in printing accuracy. We successfully printed the heart-shaped pattern and then demonstrated that it could work well. This showed that the well-defined pattern with good photoelectric properties can be obtained by using an inkjet printing process with silver nanowires ink as inkjet material.

## 1. Introduction

In recent years, silver nanowires (AgNWs) flexible transparent conductive films (FTCFs) with excellent comprehensive performance have attracted considerable attention and research because AgNWs networks can provide desirable electrical conductivity, excellent transmittance, and great flexibility [1,2,3,4,5]. However, it is highly challenging to engineer a FTCFs that can be controlled by deposition and patterned target devices using low-cost fabrication techniques [4,5,6,7,8,9]. In the past few years, many great efforts have been made to develop the preparation technology and promote the performance of AgNWs-FTCFs, including spray coating [10], gravure printing [11], screen printing [8,12], inkjet printing [9,13,14], and eletrohydrodynamic jet printing [15]. Although these methods have the advantages of simple operation, high efficiency, time efficiency, and low cost, they still present many challenges, such as poor uniformity and repeatability and controllability of samples, and the subsequent laser etching process to achieve graphics, which greatly restricts the promotion and application of the AgNWs-FTCFs in flexible devices [10,11,12,13,14,15].

Compared to the conventional silicon processing, printable electronics offer enormous potential in their ability to enter new markets as the cost of production is decreased and new solution-processable electronic materials are developed [16,17,18,19,20]. Inkjet printing process, as a printing electronic technology, is a growing technology, which is able to deposit a desired amount of materials directly from a computer-designed image onto a designed pattern of a substrate by generating drops from a reservoir with minimal human involvement [9,13,14]. It has demonstrated to be able to print all materials required for integrated circuits: conductors, semiconductors, and dielectrics, and thus transistors [21,22,23,24]. It will be one of the most promising techniques for the fabrication of AgNWs-FTCFs, due to many advantages over conventional methods such as non-contact material jetting process, maskless, little waste, design freedom, flexible or rigid substrate, etc. [9,13,14]. Although the inkjet printing technology has many advantages, nozzle clogging has always been an important problem that limits its applications because micro- or nanoparticles in the inks tend to agglomerate and precipitate during the printing process [9,13,14,23,24,25,26]. Therefore, the preparation of inkjet inks is often a challenging issue for researchers.

Depending on the inkjet printer type, the inks should have appropriate properties to jet reliably and consistently [22,23,24,25,26]. The components of conductive ink mainly include conductive fillers, solvent, adhesive phase, and functional additives, and the conductivity of printing patterns is closely related to the type and content of conductive components and additives [24,25,26]. At present, many researchers have done a lot of research works on the preparation of inkjet printing inks and the printing of functional circuits [23,24,25,26]. However, they mainly focused on nanoparticles (silver nanoparticles, graphene) [27,28] or organic materials (solution-processed poly(3,4-ethylenedioxythiophene): poly(styrenesulfonate) (PEDOT: PSS), polyaniline) [29] as conductive fillers. Recently, the inkjet-printed non-spherical noble metal nanoparticles patterns useful for drug release were reported by Chirico and coworkers [30,31], which indicated that the inkjet printing technology has a wider range of applications.

So far, only a few papers reported the preparation of the AgNWs inks and its applications in the flexible devices [9,13,14,27,32,33]. Coleman and his coworkers demonstrated the fabrication of AgNWs conductive pattern on Polyethylene terephthalate (PET) surface by inkjet printing technology, displaying conductivity as high as 10^5^ S·m^−1^ and transmittance of ~50% [9]. Lu and his coworkers reported the fabrication of the AgNWs transparent conductive networks by inkjet printing and applied them as the top electrode for inverted semi-transparent organic photovoltaic devices [13]. However, the clogging of the nozzle existed during the printing process. Huang and coworkers reported inkjet printing of AgNWs ink with a concentration of 10 mg·mL^−1^ to obtain opaque AgNWs conductors on photo paper and PET substrates, respectively [14]. It should be pointed out that the nozzle diameter of the printhead used in their work is 80 μm. Inkjet printing AgNWs flexible transparent conductive film is less reported, the main problems being: (1) AgNWs easily causes nozzle clogging; (2) low concentration of AgNWs ink results in multi-layer printing; (3) low printing accuracy; (4) wetting behavior of AgNWs ink on the substrate; (5) film homogeneity. Therefore, it is necessary to conduct in-depth and continuous research on inkjet printing AgNWs inks and AgNWs-FTCF [9,13,14,27,32,33,34].

In this work, inkjet printing AgNWs ink was prepared with AgNWs of an average diameter of 20 nm and average length of 2–5 μm as the conductive fillers dispersed into the mixed solution of 15:10:0.005 volume ratio of ethylene glycol (EG) and isopropyl alcohol (IPA) and wetting agent. Further, AgNWs-FTCF printed on PET substrate was fabricated and photoelectric performances were discussed. A well-defined pattern circuit of AgNWs networks was fabricated by using inkjet printing process, which demonstrated the application prospects of the inkjet printing process in the patterned flexible transparent electrode.

## 2. Results and Discussion

### 2.1. Sivler Nanowires Ink

Inkjet printing is a very sensitive process that depends on several factors, such as type and model of the printer, substrate, ink property, printing speed, output quality and method, pre-/post-treatment of the printed pattern, and so on. In inkjet printing, the quality of the printed pattern depends largely on the proper capability of inks, pinthead, and substrates [34,35,36]. The viscosity and surface tension of AgNWs ink are two important parameters that affect droplet morphology and size, printing adaptability, droplet spreading, and finally microstructure and performance of the printed film [35,36]. The wetting behavior of the AgNWs ink on the polyethylene terephthalate (PET) substrate is also a major factor affecting the microstructure and performance of the printed film. The solvent system has a significant effect on the nozzle clogging during the inkjet printing process, stability of ink, and the uniform film forming during the drying [9,35,36]. The microelectronic printer in our work was purchased from Shanghai Mifang Electronic Technology Co., Ltd., Shanghai, China, including an ink box with 16 nozzles with a 20 µm orifice arranged in a row. To the inkjet printer, the viscosity of the ink is the range of 2–10 mPa∙s to ensure that the ink droplets of a qualified shape are ejected. The viscosity requirement of the microelectronic printer is put forward by the equipment supplier, which is determined by the nozzle design. The AgNWs was dispersed in ethanol, but due to low viscosity, the AgNWs suspension was not printable. Considering the appropriate rheological properties of the ink and solvent evaporation during and after deposition, ethylene glycol (EG), isopropyl alcohol (IPA), ethanol, and their mixtures were used as solvent system. EG, IPA, and ethanol with different volume percentages were mixed to measure the viscosity and surface tension of the mixed solves system. The measurements for the various solvent systems were given in the Table S1 (see the Supplementary Materials for more details). With the increase of the EG volume fraction in the solvent system, the viscosity and the surface tension of the solve increase. Under the same EG volume fraction, the viscosity of IPA solvent system is higher than that of the ethanol solvent system, but the surface tension is low.

It is known that the viscosity of the ink affects the droplet’s shape, the spread of the droplet, and the final printed shape. The high viscosity ink is prone to a long tail behind the droplet head, which leads to the formation of satellite droplets. In addition, low viscosity ink tends to spray ink droplets in a water flow state. The high surface tension of the ink results in being insufficient to drive the voltage in the nozzle to eject a droplet. However, too low surface tension of the ink causes the ink spread to over the nozzle plate and cover the nozzle [33,34]. To ensure the best droplet formation and surface wetting, it is important to choose the appropriate viscosity and surface tension of ink. By comparing the viscosity and surface tension of the solvent system, 15:10 volume ratio of EG and IPA was chosen as the optimum ratio of the solvent system. Note that EG also acts as a moisturizer that prevents blocked nozzles.

To avoid clogging the nozzle, the size of the AgNWs cannot be too long and the concentration cannot be too high. The AgNWs in our work were purchased from Haitai Naxin Technology (Chengdu) Co., Ltd. The AgNWs with an average length of 2–5 µm and average diameter of about 20 nm were dispersed in the ethanol to form a 10 mg·mL^−1^ suspension. Using the ultrasonic-induced fracture process to obtain short-sized AgNWs, we found that the size distribution range of the AgNWs after fracture was large, so when the ink droplets were ejected on the PET surface, the accumulation of AgNWs was prone. However, there are some silver nanoparticles in the AgNWs suspension. One milliliter of AgNWs suspension was added into the solvent system in Table S1 to measure the viscosity, surface tension, contact angle (CA), and pH value of mixed solution. The measurements for the various mixed solutions are given in Table S2 (see the Supplementary Materials for more details). The viscosity and surface tension of the mixed solution decreased after 1 mL of AgNWs ethanol suspension was added. To the mixed solvent system, the CA between the droplet and the PET surface decreased obviously with the decrease of the volume fraction of EG. The pH values of all of mixed solutions were weak alkaline. By comparing the viscosity, surface tension, CA, and pH value of the mixed solutions, sample No. 1, namely 10 mL EG, 10 mL IPA, and 1 mL AgNWs suspension, was chosen as the formula system of ink.

However, for excellent ink droplet spreading on the PET substrate, good wetting behavior or low CA of the ink on the PET substrate is necessary [34,35,36]. The CA of the above formula system of ink on the PET substrate surface was large, so polyether-modified polysiloxane (Silcona 137) as a wetting agent was added into the above mixed solution. The molecular structure of the Silcona 137 contains both hydrophilic polyether segments and hydrophobic polysiloxane segments, which improves the wettability of ink on the surface of the PET substrate. Ten microliters of Silcona 137 was added into the solution system in Table S2 to measure the viscosity, surface tension, CA and pH value of the solution. The measurements for the various solutions are given in the Table 1. The viscosity of the mixed solution and the CA between the ink droplet and the PET surface decreased after adding 10 μm of Silcona 137, which had little effect on the surface tension and the pH value.

By comparing the viscosity, surface tension, CA, and pH value of ink, sample No. 1, namely 10 mL EG, 10 mL IPA, 10 µL of Silcona 137, and 1 mL AgNWs suspension whose viscosity, surface tension, CA and pH value of the AgNWs ink were 7.1 mPa∙s, 29.583 mN·m^−1^, 22.5°, and 7.88, respectively, was selected to continue adjusting the formula. Other parameters remained unchanged, reducing the amount of the Silcona 137 to 5 µL, and the viscosity, surface tension, CA, and pH value of the AgNWs ink were 7.4 mPa∙s, 29.581 mN·m^−1^, 24.5°, and 7.76, respectively. In order to obtain high photoelectric performance of AgNWs films as much as possible, we considered choosing a formula with low Silcona 137, so the AgNWs ink formula was 0.38 mg·mL^−1^ AgNWs in the mixture of 15:10:0.005 volume ratio of EG and IPA and Silcona 137. We did not observe the obvious deposits after the above AgNWs ink was placed in the refrigerator (8 °C) for 2 months. After being injected into the ink cartridge by ultrasonic dispersion, ink droplets were still able to be ejected smoothly, indicating that the formulated AgNWs ink has good dispersion stability.

**Figure 1:**
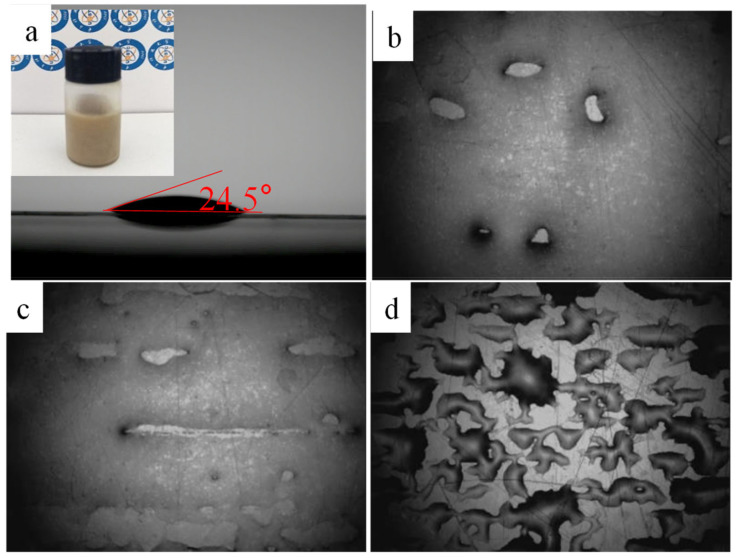
Contact angle image of ink droplet (**a**) and photographs of the ink on the PET surface after printing a layer (taken by the ink-et printer’s built-in camera) with nozzle spacing of 10 µm (**b**), 15 µm (**c**), and 20 µm (**d**).

The formulated AgNWs ink was inkjet-printed on a clean PET surface. Inkjet printing parameters were as follows: voltage of 20 V; number of nozzles of 16; drop frequency of 7000 Hz; and ink droplet spacing of 10 μm, 15 μm, and 20 μm, respectively. During the printing process, the PET was heated to 40 °C and the nozzles were heated to 35 °C, attempting to enhance the fluidity of AgNWs ink to prevent clogging of nozzles. The printed AgNWs films were immediately dried on a heater at 80 °C for 10 min after printed. Figure 1 shows the CA image of ink droplet (Figure 1a) and photographs of the ink on the PET surface after printing a layer (taken by the inkjet printer’s built-in camera) with droplet spacing of 10 µm (Figure 1b), 15 µm (Figure 1c), and 20 µm (Figure 1d), respectively. Inserted in Figure 1a is a photograph of AgNWs ink. When the droplet spacing was 10 μm, there were a few blank areas in the liquid film, indicating that drops overlap and merge; when the droplet spacing was 15 μm, the blank area in the liquid film increased; when the droplet spacing was 20 μm, the droplets failed to connect and blend together and isolated drops landed with large spacing. To ensure that the ink droplets could be spread and connected to each other to form a liquid film and avoid an overflowing irregular bead forms, we chose ink droplets spacing of 15 μm.

**Figure 2:**
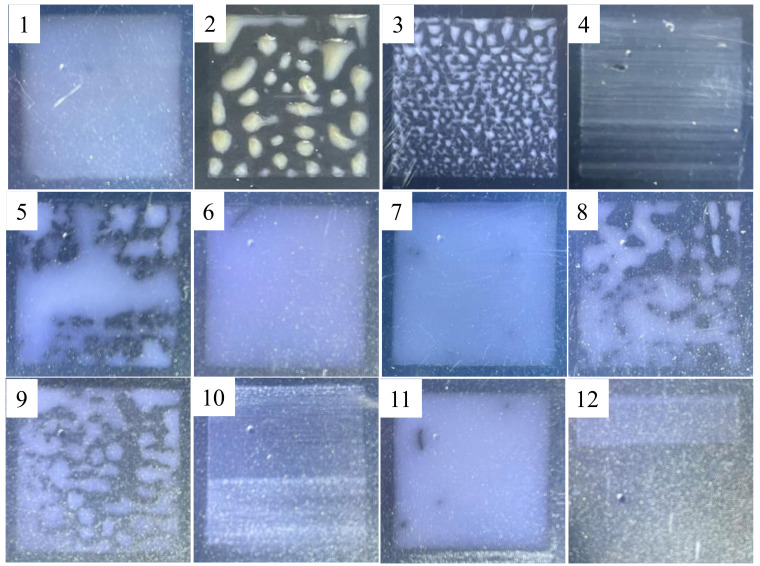
Photographs of the wet liquid film printed once with AgNWs inks in Table 1.

Choosing the ink droplet spacing of 15 µm, and other printing parameters as above, all the formula AgNWs inks in Table 1 were printed on the PET surface once respectively, and then photographs of the wet liquid film were taken, as shown in Figure 2. The viscosities of the AgNWs inks of 2, 3, 8, and 9 were suitable for printing, but due to the small CA between the droplet and the PET surface, the ink droplets were too far apart to interact, and then isolated droplets landed; the CA between the droplet of the AgNWs ink of 5 and the PET was 32.5°, but the viscosity of the ink was too high to eject smoothly from the nozzle, and it could also be that because of the same drive voltage, the squeeze pressure applied to the ink with high viscosity after the piezoelectric ceramic deformation was not enough to overcome the ink surface tension and gravity, resulting in less ejection of ink [34]. The AgNWs inks of 4, 10, and 12 have low viscosities, where the CA between the ink droplets of 4 and 10 and the PET are 2.0°, and 8.5° for the ink of 12, and three kinds of inks were printed on the surface of PET to form the thin liquid films, which may be related to the low viscosity ink in the form of small droplets rapidly jetting and flowing out from the nozzle. The AgNWs inks of 1, 6, 7, and 11 can form liquid film on the surface of PET, respectively, apparently caused by droplet spreading and blending into each other. The viscosity of ink of 6 is too high and is not suitable for the equipment.

### 2.2. Properties of Ink-Jet Printed AgNWs Films

**Figure 3:**
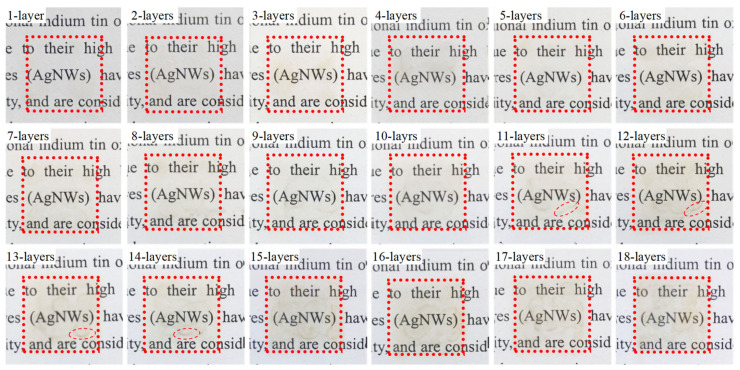
Photographs of printed AgNWs films with 1–18 layers after heat treatment at 80 °C for 10 min, respectively, and the red dotted box is 2 cm × 2 cm in size.

The formulated AgNWs ink (0.38 mg·mL^−1^) was printed on 2 cm × 2 cm square film on a PET substrate by microelectronic printer. Inkjet printing parameters were as follows: voltage of 20 V, number of nozzles of 16, drop frequency of 7000 Hz, and ink droplets spacing of 15 μm. During the printing process, the PET substrate was heated to 40 °C and the nozzles were heated to 35 °C. Figure 3 presents photographs of printed films with 1–18 layers after heat treatment at 80 °C for 10 min, respectively, and the size of the red dotted box was 2 cm × 2 cm in size. It is clear that with the increase of the printed layer, the deposition of AgNWs increases on the PET surface, which results in the optical transmittance of the film gradually decreasing. However, the words on the background are clearly visible, indicating that the light transmittance of the film is good. No obvious accumulation or non-uniformity of AgNWs on the surface of the thin film with 1–10 layers was observed by naked eye. The tiny lines (red dotted ellipses) formed by the accumulation of AgNWs can be observed on the film of 11–14 layers films. No obvious accumulation of AgNWs was observed on the 15–18 layer films, indicating that AgNWs were uniformly distributed on the surface of PET substrate in general. It also indicated that the formula of the AgNWs ink was conducive to the ink droplet spreading on the surface of the PET and the distribution of AgNWs before drying.

**Figure 4:**
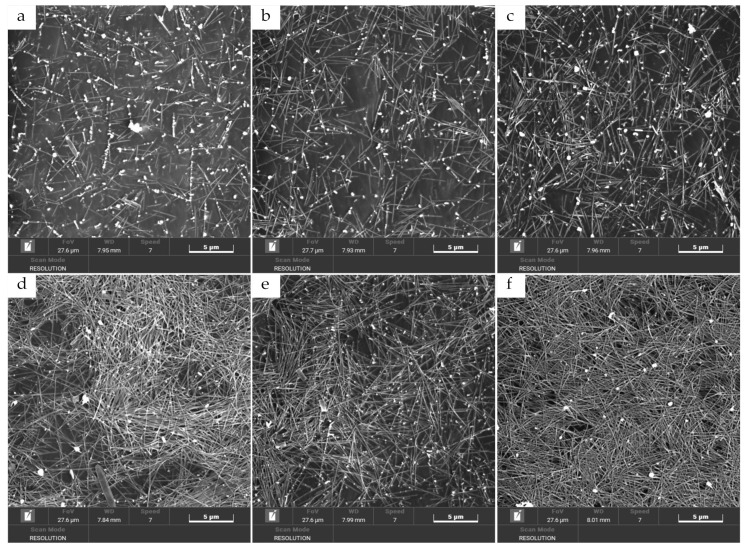
SEM images of AgNWs fims with the printing layers of 3 (**a**), 6 (**b**),9 (**c**), 12 (**d**), 15 (**e**), and 18 layers (**f**), respectively.

In order to analyze the distribution of AgNWs and the micromorphology of AgNWs films with different layers, we characterized SEM images of AgNWs films with 1 (Figure 4a), 6 (Figure 4b), 9 (Figure 4c), 12 (Figure 4d), and 15 layers (Figure 4e), as shown in Figure 4. As can be seen here, the AgNWs deposited by inkjet printing are uniformly distributed on the surface of the PET substrate as a whole and only a small amount of local accumulation of the AgNWs was observed. It should be pointed out that the PET substrate was not specially treated, indicating that the uniform distribution of the AgNWs could be due to the good wettability between the AgNWs ink droplets and the PET substrate surface. Only a small amount of the AgNWs was deposited in a scattered matter on the surface of PET after printing one layer of the film. The network structures of the AgNWs that overlapped with each other are visible on the six-layer printed film. On the 15-layer and 18-layer films, not only can the multi-layer AgNWs be stacked and deposited on the entire PET surface, but also enhance the uniformity of the AgNWs distribution.

Lu and coworkers reported, for the first time, to prepare inkjet-printed AgNWs networks as the top electrode for inverted semi-transparent organic photoltaic devices and observed the distinct non-uniform distribution of AgNWs and AgNWs aggregation, which was due to the poor wettability between the AgNWs ink and anode buffer layer [13]. Coleman and coworkers reported that the well-defined patterns were fabricated with AgNWs networks by inkjet printing [9]. Although the distribution of AgNWs is uniform, the patterns were semi-transparent. Compared to the previous literatures, the main reason for obtaining good optical transmittance is related to the presence of Silcona 137 in our ink formulation that significantly improves the wetting and spreading of ink droplets on the PET surface.

**Figure 5:**
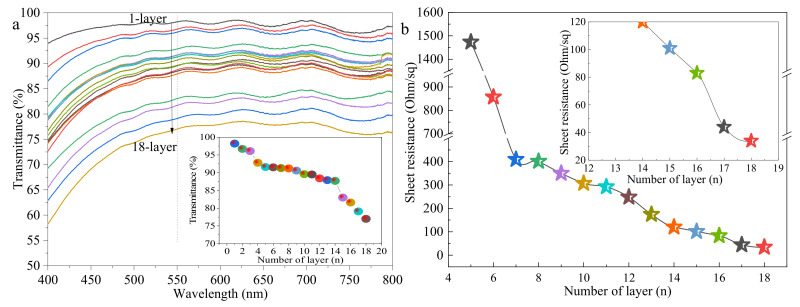
Optical transmittance (**a**) and (**b**) sheet resistances of samples with different printed layers. The inserts are optical transmittance at 550 nm in (**a**) and local magnification in (**b**).

Figure 5 displays the optical transmittance in the range of 400–800 nm and sheet resistance of the printed AgNWs films with 1–18 layers (as shown in Figure 2). We observed that the transmittance of AgNWs films decreases slightly from 98.2% to 87.7% with the increase of printed layers from 1 to 14 and decreases sharply to 77.0% with an increase of printed layers to 18. The AgNWs films below four layers are nonconductive. The sheet resistance of printed AgNWs films decreases dramatically from 1473 Ω∙sq^−1^ to 409 Ω∙sq^−1^ with the increase of the printed layers from 5 to 7, and decreases gradually to 34 Ω∙sq^−1^ with the increase of printed layers to 18. Note that the AgNWs films with printed layers of 16 and 17 have average sheet resistance of 83 Ω∙sq^−1^ and 44 Ω∙sq^−1^ and average transparency of 81.9% and 79.8%, respectively, which meet the requirements of the flexible transparent conductivity film for photoconductive properties. In fact, we measured the optical transmittance and absorbance in the range of 400–2400 nm, which is shown in Figure S1. The light transmittance of films with 1–8 layers is not obviously changed in the range of 400–2400 nm. However, when the number of printing layers exceeds 8, the light transmittance in the range of 800–2400 nm gradually decreases, and with the increase of the number of printing layers, the decrease in light transmittance becomes obvious. Meanwhile, the absorption of the AgNWs film slightly increases in the near-infrared region, however, the light absorption in the range 400–2400 nm is relatively weak, and the light absorption of the 18-layer film is less than 0.4%.

Previous research literatures showed that AgNWs with high length–diameter ratio are beneficial to obtaining films with high photoelectric properties [1,2,3,4]. However, for inkjet printing processes, the AgNWs with a high length-diameter ratio are not applicable due to limited nozzle size. It should be noted that we can obtain available AgNWs films with suitable photoelectric properties by the inkjet printing process, showing the feasibility and potentiality of the technology application in the field of printing electronics.

### 2.3. Optimization of Heat Treatment Temperature

**Figure 6:**
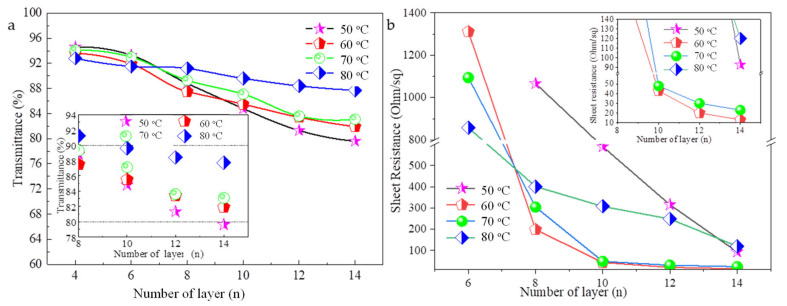
Transmittance at 550 nm (**a**) and sheet resistance (**b**) of the AgNWs films with different printed layers under different heat treatment temperatures. The inserts are local magnification.

When the ink droplet impacts the PET substrate, it deforms and dries, and the drying process has a significant effect on the obtained printed film morphology [34]. Using the inkjet process parameters described above, the AgNWs films of 2 cm × 2 cm in size with 4, 6, 8, 10, 12, and 14 layers were printed and then heat treated at 50 °C, 60 °C, 70 °C, and 80 °C, respectively. The heat treatment time required 20 min at 50–70 °C and 10 min at 80 °C. Figure 6 shows optical transmittance at 550 nm (Figure 6a) and sheet resistance (Figure 6b) of the AgNWs films with different printed layers under different heat treatment temperatures. The inserts are local magnification. When the number of printing layers is 4–8, the transmission of films with heat treatment at different temperatures is similar; when the number of printing layers is 10–14, the transmittance of the film increases as the heat treatment temperature increases. The transmittance of 14-layer AgNWs films increases from 79.6% to 87.7% as the heat treatment temperature increases from 50 °C to 80 °C. The printed six-layer AgNWs film cannot test the sheet resistance after heat treatment at 50 °C. While, the heat treatment temperatures increase to 60 °C, 70 °C, and 80 °C, the sheet resistances of films reach 1311 Ω∙sq^−1^, 1094 Ω∙sq^−1^, and 857 Ω∙sq^−1^, respectively. However, as the number of printing layers continues to increase, the sheet resistances of films with heated treatment at 60 °C and 70 °C are significantly lower than those of heat treatment at 50 °C and 80 °C. Note that the AgNWs films with 10 and 12 layers with heated treatment at 60 °C and 70 °C have average sheet resistances of 43 Ω∙sq^−1^ and 48 Ω∙sq^−1^ and average transparency of 85.5% and 87.1%, respectively, which are close to that of the widely used ITO electrode; and the AgNWs film with 14 s layers with heat treatment at 60 °C and 70 °C has an average sheet resistance of 13 Ω∙sq^−1^ and 23 Ω∙sq^−1^ and average transparency of 81.9% and 83.1%, respectively, which displays good photoelectric performance. Photographs of printed AgNWs films with different layers of heated treatment at 50 °C, 60 °C, and 70 °C are shown in Figure S2. From the naked eye observation, the film formation quality heated treatment at 50 °C is not worse than that of heat treatment at 60–80°.

**Figure 7:**
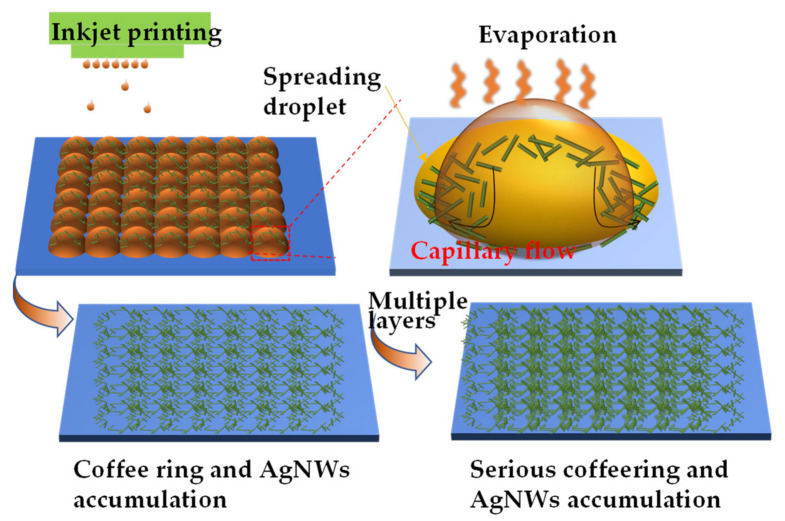
Schematic diagrams of capillary flow induced coffee ring and AgNWs accumulation.

The temperature and time of the heat treatment required for the liquid film are related to the solute properties. When the ink droplet impacts the substrate, the droplet is deformed and spreads to a diameter that is several times its initial droplet diameter and forms the shape of thick edges and a thin center due to the external deflection of momentum [34,35]. The solvent evaporation rate relates to the Marangoni flow and capillary flow during the spreading and evaporation of the solvent. When the Marangoni flow is balanced with the capillary flow, the deposition of the AgNWs is balanced with the evaporation of the solvent, forming a uniform AgNWs distribution. On the contrary, when Marangoni flow is unbalanced with capillary flow, the deposition of AgNWs is unbalanced with solvent evaporation, causing AgNWs to easily deposit at the edge of the droplet, forming a coffee ring or AgNWs accumulation phenomenon [34,35,36], as shown in Figure 7. This phenomenon could be serious as the number of printed layers increases. Here, at a low heat treatment temperature, the solvent in the film is not fully removed, resulting in a high sheet resistance of the film; a high heat treatment temperature, results in non-uniform distribution of AgNWs, so both the electrical conductivity and light transmittance of film are poor. We need to take great care for controlling solvent evaporation.

### 2.4. Optimization of Print Frequency

**Figure 8:**
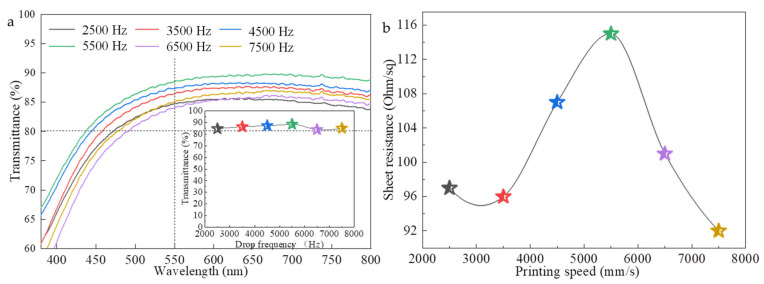
Transmittance (**a**) and sheet resistance (**b**) of the AgNWs films with different drop frequencies. The insert is transmittance at 550 nm.

In order to discuss the effect of drop frequency on the photoelectric properties of AgNWs films, the AgNWs film of 2 cm × 2 cm in size and printed layers of 10 was printed and then heat treated at 60 °C for 20 min, with drop frequencies of 2500, 3500, 4500, 5500, 6500, and 7500 Hz, respectively; other inkjet process parameters are the same as Figure 5. Figure 8 shows the optical transmittances (Figure 8a) and sheet resistances (Figure 8b) of samples. The inserts are local magnification. With the drop frequency increases from 2500 to 5500 Hz, the transmittance of AgNWs films increases slightly from 84.7% to 88.5%. After that, the transmittance of AgNWs film decreases with the increase of the drop frequencies. For the sheet resistance of the film, the same trend is observed. The printed AgNWs films at the drop frequency of 5500 Hz and 7500 Hz have a maximum sheet resistance of 115 Ω∙sq^−1^ and minimum sheet resistance of 92 Ω∙sq^−1^, respectively. The above experimental results show that the drop frequency is also a factor affecting the distribution of AgNWs, and then affecting the photoelectric performance of the AgNWs films, but it is not a key factor. The optimal drop frequency is 7500 Hz for our work. Soltman and coworkers reported that the evaporation time of the droplet was less than the drop jetting period, with each landing drop dried individually regardless of overlap, leading to what looks like offset stacked coins [34]. By carefully optimizing the drop frequency, both the microstructure and photoelectric properties of the film can be improved. In addition, increasing the drop delay might cause nozzle blockage.

**Figure 9:**
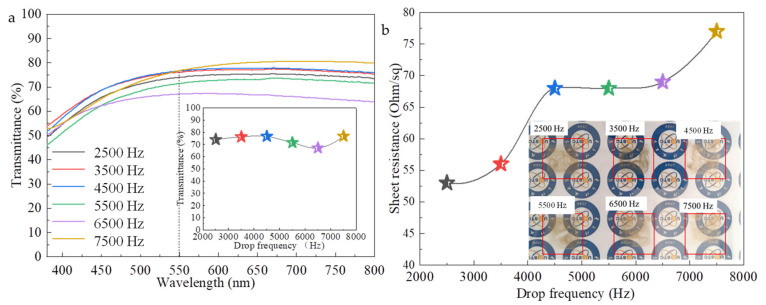
Transmittance (**a**) and sheet resistance (**b**) of the AgNWs films with different drop frequencies. The insert has transmittance at 550 nm in (**a**) and photographs of samples in (**b**).

To further understand the effect of the drop frequency on the microstructure and photoelectric properties of the AgNWs films, we increased the concentration of the AgNWs ink by two times, which is 0.74 mg∙mL^−1^, and printed the films with six layers on the PET substrate at drop frequencies of 2500, 3500, 4500, 5500, 6500, and 7500 Hz, respectively, with other inkjet process parameters kept constant. Seen from here, increasing the ink concentration, the light transmittance of the AgNWs film decreases significantly, the conductivity is significantly enhanced, and the sheet resistance of six-layer AgNWs film printed at 2500 Hz drop frequency is 53 Ω∙sq^−1^, which is about 16 times lower than that of the sample in Figure 5b. The effect of the droplet frequency on the film light transmittance is similar to Figure 8, however, as the droplet frequency increases, the sheet resistance of the film increases. Seen from photographs inserted in Figure 9b, the accumulation of AgNWs is clearly visible. This shows on the one hand that high concentration is not conducive to obtaining uniform AgNWs films, and on the other hand that high droplet frequencies are not suitable for high concentration inkjet ink systems.

### 2.5. Optimization of Number of Nozzle

**Figure 10:**
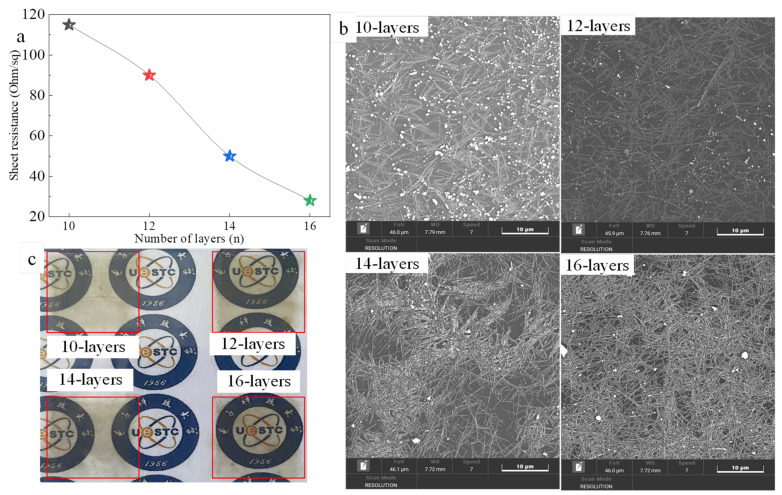
Sheet resistance (**a**) and SEM images (**b**) and photographs (**c**) of the AgNWs films with the printing layers of 10, 12, 14, and 16.

The above-printed AgNWs films used 16 nozzles to improve printing efficiency. Here, we printed AgNWs films with one nozzle to discuss the effect of the number of nozzles on the photoelectric properties of AgNWs films, and other inkjet process parameters are the same as Figure 7 except for a heat treatment temperature of 60 °C for 20 min. Figure 10 shows sheet resistance (Figure 10a) and SEM images (Figure 10b) and photographs (Figure 10c) of the AgNWs films with the printing layers of 10, 12, 14, and 16. Compared with Figure 6b, the sheet resistance is high, 2.4 times higher for the 10-layer film and 2.2 times higher for the 14-layer film. However, the films printed with one nozzle display good transmittance and uniform distribution of AgNWs as shown in Figure 10b,c. In addition, the sheet resistances of films printed with one nozzle approximately linearly decrease with the increase of number of layers. When the number of nozzles is greater than 1, the ink droplets of each layer deposited on the PET substrate of the adjacent nozzles overlap and merge, but retain individual rounded contact lines. In this case, multi-nozzle printed film thickness is greater than that of one nozzle printed, so the electrical conductivity is high, but it might cause non-uniform deposition of AgNWs due to the presence of more AgNWs.

### 2.6. Printing Accuracy

**Figure 11:**
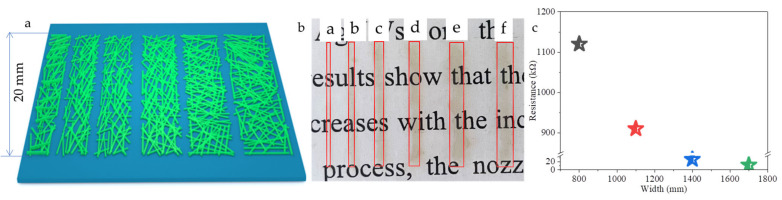
Schematic (**a**) and photographs (**b**), and resistance (**c**) of AgNWs patterns with a size of 20 mm and width of 200 μm, 500 μm, 800 μm, 1100 μm, 1400 μm, and 1700 μm, respectively.

For integrated circuits, ideal inkjet-printed lines would be smooth, even, narrow, and straight. However, previous works demonstrated a need for improved control of the behavior of inkjet-printed silver nanoparticles inks [22,23,24,25]. Here we discussed printing accuracy of AgNWs films by inkjet printing process. We designed and printed six linear patterns with lengths of 20 mm and widths of 200 μm, 500 μm, 800 μm, 1100 μm, 1400 μm, and 1700 μm, respectively, and each with 20 printed layers. Inkjet printing parameters were as follows: voltage of 20 V, number of nozzles—1, drop frequency of 7000 Hz, and droplets spacing of 15 μm. During the printing process, the PET substrate was heated to 40 °C, the nozzles were heated to 35 °C, and film was heated at 60 °C for 20 min. Figure 11 shows the designed patterns (Figure 11a); photographs of the printed patterns (Figure 11b) with 200 (curve a), 500 (curve b), 800 (curve c), 1100 (curve d), 1400 (curve e), and 1700 μm (curve f) after heat treatment; and the sheet resistances of pattern lines with different widths. It is obvious that the pattern lines with widths of 1400 μm and 1700 μm show accumulation and overflow of AgNWs at the edges, respectively. The pattern lines with widths of 200 μm and 500 μm could not test the resistance in the middle of the line, and high-resistance values can only be tested in some local areas of the edge. The resistances of other pattern lines are high; the resistance of the pattern line with a width of 1700 μm is about 3.09 KΩ.

**Figure 12:**
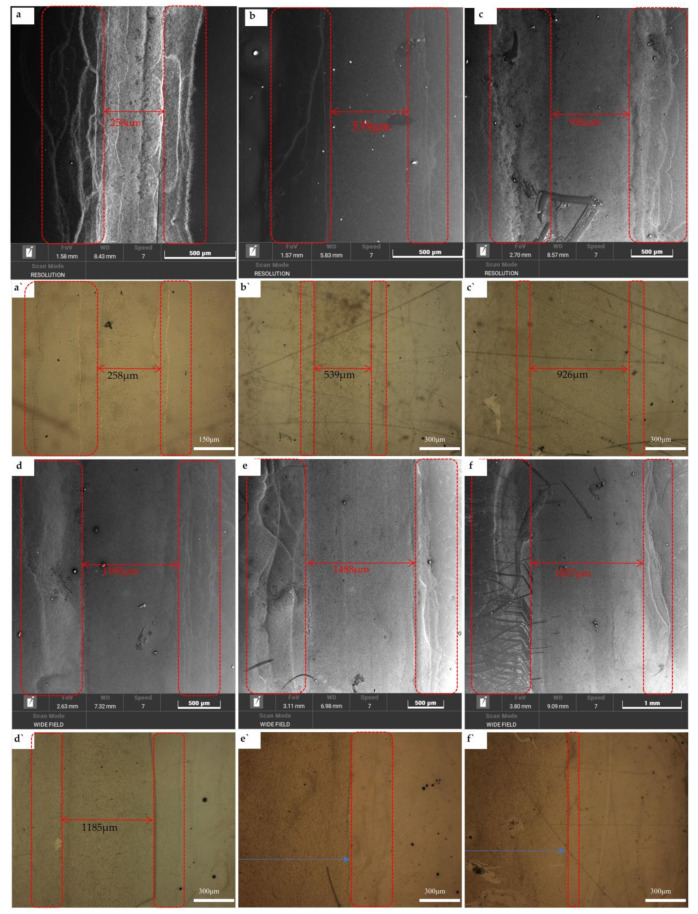
SEM images of samples in Figure 11b from (**a**–**f**), and corresponding optical microscope photographs from (**a′**–**f′**).

Figure 12a–f shows SEM images of those in Figure 11b from the printed patterns of a–f, and Figure 12a′–f′ are the corresponding optical microscope photographs. The real widths of the patterns on the PET substrate with 200 μm, 500 μm, 800 μm, 1100 μm, 1400 μm, and 1700 μm are 258 μm, 539 μm, 926 μm, 1185 μm, 1488 μm, and 1857 µm; and the difference between the real value and the design value are 58 μm, 39 μm, 126 μm, 85 μm, 88 μm, and 157 µm, respectively. Comparing Figure 2 (pattern with 2 cm× 2 cm), the inkjet-printed AgNWs narrow linear patterns are prone to the phenomenon of AgNWs accumulation and overflow of both the edges due to the presence of coffee rings [9,32,33]. For the thin linear pattern, heat is readily transferred from the substrate to the thin pinned edge of the drop, leading to enhanced evaporation near the drop’s edge compared to that at the center [34,35]. It means that the effect of temperature on coffee ring formation is enhanced. Hence, the droplet displays a greater transfer of solute to its edge when subjected to heating.

We also made six linear patterns with the same structures as Figure 11 using inkjet printing with 16 nozzles. The experiments found that the difference between the real value and the design value is much greater than that of one nozzle, indicating that the ink droplets are spread and overlap each other, which will lead to a more serious ink overflow in the case of the multi-nozzle printing.

### 2.7. Applications of Inkjet Printting Patterns

**Figure 13:**
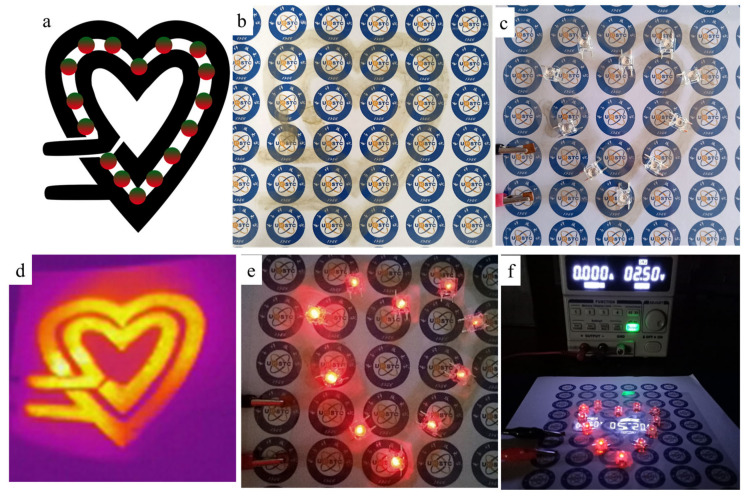
Designed pattern with a circuit (black) and LED beads (circles) (**a**), photographs of printed pattern before (**b**) and after (**c**) heat treatment, infrared thermal imaging (**d**), lighting on the LED beads (**e**), and applied direct current (DC) voltage of 2.5 V (**f**).

**Figure 14:**
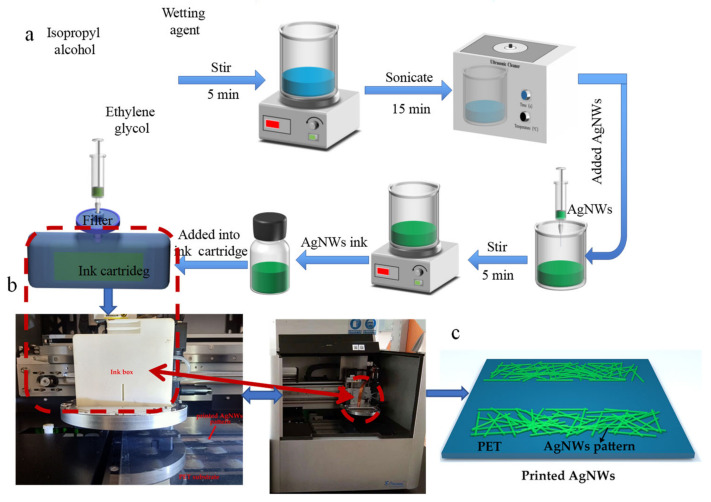
Schematic diagrams of the preperation of AgNWs ink (**a**) and inkjet printing equipment (**b**) and inkjet printed AgNWs patterns (**c**).

We designed and printed a heart-shaped circuit pattern on the PET substrate with 4 cm × 4 cm in size using AgNWs ink. The printed layers were 14 layers. The heart-shaped line pattern and the light emitted diode (LED) beads (0.2 W per) were assembled into a circuit. Figure 13 shows designed pattern with a circuit (black) and LED beads (circles) (Figure 13a), photographs of printed pattern before (Figure 13b) and after (Figure 13c) heat treatment, infrared thermal imaging (Figure 13d), lighting on the LED beads (Figure 13e), and applied direct current (DC) voltage of 2.5 V (Figure 13f). Seen from Figure 14b,c, the printed heart-shaped line circuit pattern has a good shape before and after heat treatment. The infrared thermal imaging shows a uniform heat distribution over the whole pattern, indicating that the distribution of the AgNWs on the surface of the PET is generally uniform. Meanwhile, all of the LEDs worked well (Figure 13e,f). These demonstrated that the well-defined AgNWs pattern with good photoelectric properties can be obtained by using the inkjet printing process. Of course, further improvements, such as improving efficiency and photoelectric performances and the accuracy of line, etc., are still necessary. We believe that our research works play an important role in promoting inkjet printing processes in preparing flexible AgNWs transparent electrodes or circuits with well-defined patterns.

## 3. Materials and Methods

### 3.1. Materials

Silver nanowires of about 20 nm in diameter and 2–5 μm in length were purchased from Haitai Naxin Technology (Chengdu) Co., Ltd., Chengdu, China, as a suspension in ethanol at a concentration of 10 mg·mL^−1^. Isopropyl alcohol (IPA ≥ 99.7%) was purchased from Tianjinshi Baishi Chemical Co., Ltd., Tianjin, China. Ethylene glycol (EG) was purchased from Tianjin Yongda Chemical Reagent Co., Ltd., Tianjin, China. PET as a substrate was purchased from Dinglishen New Materials Co., Ltd., Zhongshan, China. Polyether-modified polysiloxane (Silcona 137) as a wetting agent was purchased from Oncell Co., Ltd., Guangzhou, China. All the chemicals were used as received.

### 3.2. Methods

#### Preparation of Silver Nanowires Ink and Flexible Transparent Film 

Wetting agent (Silcona 137) of 5mL and isopropanol of 10 mL were added into ethylene glycol of 15 mL and magnetically stirred for 5 min and then ultrasound for 15 min. AgNWs suspension of 1 mL was added into the above-mixed solution and stirred for 5 min to obtain AgNWs ink with a concentration of 0.38 mg·mL^−1^. A microelectronic printer (Shanghai Mifang Electronic Technology Co., Ltd., Shanghai, China) was used in our work, which includes a single printhead having 16 nozzles with diameters of 20 μm, driven by piezoelectric elements jetting. The number of nozzles used can be controlled using BitsAssembler (software for controlling microelectronic printer) and each nozzle can be controlled via each piece of piezoelectric ceramic. At first, we designed a square of 2 cm × 2 cm by BitsAssember. PET substrate was cleaned with deionized water and ethanol successively, and then vacuum-adsorbed on the platform of the inkjet printer. The AgNWs ink was injected into the cartridge of the inkjet printer and then the film was printed. The inkjet printing parameters were as follows: voltage of 20 V, number nozzles of 16, droplet frequency of 7500 Hz, and droplet spacing of 10 μm. The jetting waveform parameters are shown in Figure S2 (Supplementary Materials), which was recommended by the equipment manufacturer. For controlling solvent evaporation during printing and after deposition, the PET substrate temperature was kept constant at 40 °C and the nozzles kept at 35 °C during printing. After one layer was printed, the film was heated at 80 °C for 10 min and then cooled to 40 °C to print the next layer. The flexible transparent conductive films with different printed layers were finally obtained. Figure 14 shows schematic diagrams of the fabrication of the AgNWs conductive ink and AgNWs patterns.

### 3.3. Characterizations

A digital viscometer (NDJ-1S, Shanghai Qili Scientific Instrument Co., Ltd., Shanghai, China) was used to measure the viscosity of AgNWs ink. An automatic tension meter (JK99C, Shanghai Zhongchen Digital Technology Equipment Co., Ltd., Shanghai, China) was employed to measure the surface tension of AgNWs ink, and the contact angle measurement (JC2000C1, Shanghai Zhongchen Digital Technology Equipment Co., Ltd., Shanghai, China) was chosen to measure the contact angle of the ink on PET. A sheet resistance meter (DMR-1C, Nanjing Daming Instruments Co., Ltd., Nanjing, China) was utilized to measure the sheet resistance of the flexible transparent conductive film, a haze meter (TH-100, Hangzhou Caipu Technology Co., Ltd., Hangzhou, China) was used to measure the haze value, and a spectrophotometer (UH415 UV, Beijing Techcomp Scientific Instrument Co., Ltd., Beijing, China) was performed to measure the relationship between wavelength and light transmittance. A scanning electron microscope with a digital camera (SEM, Zeiss sigma 500, Carl Zeiss, Germany) and an optical microscope (Nikon LV100, Nikon Co., Ltd., Tokyo, Japan) were used to characterize the microstructures of the AgNWs flexible transparent conductive film. An infrared thermal imaging camera (UTI160G, range: −20–350 °C, accuracy: ±2 °C, UNI-T China Co., Ltd., Shenzhen, China) was used to take infrared thermal images. A regulated DC power supply (DPS-3010D, Shenzhen Zhaoxin Electronic Equipment Co., Ltd., Shenzhen, China) was used as the driving power supply.

## 4. Conclusions

In summary, the effects of the solvent system, wetting agent, AgNWs suspension on the viscosity, surface tension, contact angle between ink droplet and PET surface, and pH value of AgNWs ink were discussed. AgNWs ink of a concentration of 0.38 mg·mL^−1^ was considered as an optimized formula for printing AgNWs film, which was obtained from silver nanowires dispersed into a mixed solution of 15:10:0.005 volume ratio of EG and IPA and wetting agents. The effects of the number printing layer, heat treatment temperature, print frequency, and number of nozzle on the microstructures and photoelectric properties of AgNWs films were investigated in detail. The experimental results showed that the printed 14-layer AgNWs film heated at 60 °C or 70 °C has average sheet resistance of 13 Ω∙sq^−1^ or 23 Ω∙sq^−1^ and average transparency of 81.9% or 83.1%, respectively, which displayed good photoelectric performance when the inkjet printing parameters were set to the voltage of 20 V, number of nozzles—16, drop frequency of 7000 Hz, ink droplets spacing of 10 μm, PET substrate temperatures of 40 °C and nozzles of 35 °C during printing, and heated treatment at 60 °C for 20 min. The printing accuracy of the patterns was studied using printed lines with different widths. The accumulation and overflow of AgNWs at the edges were observed, which results in a decrease in printing accuracy. We designed a heart-shaped pattern 4 cm × 4 cm in size and printed it on the surface of the PET using AgNWs ink, and then it was assembled into a circuit with LED beads. All of the LEDs worked well, which demonstrated that the well-defined AgNWs pattern with good photoelectric properties can be obtained by using the inkjet printing process. Further improvements, such as improving the efficiency and photoelectric performances, and the accuracy of line, etc., are still necessary. We believe that our research works play an important role in promoting inkjet printing processes in preparing flexible AgNWs transparent electrodes or circuits with well-defined patterns.

## Figures and Tables

**Table 1:** Viscosity and surface tension and CA and pH value of the AgNWs inks with different formulations (at 28 °C).

No.	Ethylene Glycol (mL)	Isopropyl Alcohol (mL)	Ethanol (mL)	Silcona 137 (μL)	AgNWs (mg·mL^−1^)	Viscosity (mPa·s)	Surface Tension (mN·m^−1^)	Contact Angle (°)	pH
1	15	10	0	10	1	7.1	29.583	22.5	7.88
2	10	15	0	10	1	5.3	26.175	15.0	7.88
3	5	20	0	10	1	3.5	23.708	9.0	8.12
4	0	25	0	10	1	2.2	22.167	2.0	7.98
5	25	0	0	10	1	13.8	33.083	32.5	8.24
6	20	5	0	10	1	10.9	31.792	30.5	8.19
7	15	0	10	10	1	5.4	31.375	23.5	8.07
8	10	0	15	10	1	3.3	27.792	15.0	7.86
9	5	0	20	10	1	2.0	24.992	10.5	8.25
10	0	0	25	10	1	1.8	22.833	8.5	8.09
11	20	0	5	10	1	8.6	35.417	35.0	20
12	0	20	5	10	1	1.9	22.042	2.0	0

## Data Availability

Data sharing not applicable. No new data were created or analyzed in this study. Data sharing is not applicable to this article.

## Supplementary Materials

The following are available online at https://www.mdpi.com/article/10.3390/ijms22147719/s1.
