# Supplementary material for: Novel Insights into Inkjet Printed Silver Nanowires Flexible Transparent Conductive Films
Source: Int J Mol Sci. 2021 Jul 19;22(14):7719. doi: 10.3390/ijms22147719 (PMC8307527; doi:10.3390/ijms22147719)
Supplement: Supplementary file 1 [file ijms-22-07719-s001.zip › ijms-1283448-supplementary.pdf]

Supporting Information

Novel Insights into Inkjet Printed Silver Nanowires Flexible Transparent Conductive Films

Yuehui Wang[1,*], Xiaoli Wu[1,2], Ke Wang[1], Kaiwen Lin[1,*], Hui Xie[1], Xiaobing Zhang[1], and Jing-Ze Li[2,*]

[1] Department of Materials and Food, University of Electronic Science and Technology of China Zhongshan Institute, Zhongshan528402, China; wangzsedu@126.com (Y.W.); 201921030315@std.uestc.edu.cn (X. W.); wangkezsedu@126.com (K.W.); kevinlin1990@163.com (K. L.); Xiehuizsedu@126.com (H.X.); semiben@foxmail.com (X. Z)

[2] School of Materials and Energy, University of Electronic Science and Technology of China, Chengdu 610054, China; lijingze@uestc.edu.cn (J-Z.L.)

* Correspondence: wangzsedu@126.com (Y.W.); kevinlin1990@163.com (K.L.); lijingze@uestc.edu.cn (J-Z.L.); Tel.: +86-760-8832-5402

Table S1 Viscosity and surface tension of different solve systems (at 28 °C)

No.	Ethylene glycol（mL）	Isopropyl alcohol（mL）	Ethanol（mL）	Viscosity (mPa·s)	Surface tension ($mN·m^{-1}$)
1	15	10	0	8.8	29.958
2	10	15	0	5.1	25.750
3	5	20	0	3.5	24.250
4	0	25	0	2.3	21.708
5	25	0	0	14.0	42.083
6	20	5	0	10.6	34.083
7	15	0	10	5.4	33.833
8	10	0	15	3.1	26.792
9	5	0	20	1.9	25.250
10	0	0	25	1.2	22.708
11	20	0	5	8.7	35.167
12	0	20	5	2.1	21.750

Table S2 Table 1 Viscosity and surface tension and CA and pH value of the AgNWs inks with different formulations (at 28°C)

No.	Ethylene glycol（mL）	Isopropyl alcohol（mL）	Ethanol（mL）	AgNWs (mg·mL^{-1})	Viscosity (mPa·s)	Surface tension (mN·m^{-1})	Contact Angle (°)	pH
1	15	10	0	1	7.6	29.580	35.0	7.45
2	10	15	0	1	4.9	25.667	17.0	7.92
3	5	20	0	1	3.3	23.458	9.5	8.17
4	0	25	0	1	2.3	21.427	8.0	7.35
5	25	0	0	1	13.2	40.458	45.5	7.68
6	20	5	0	1	10.6	30.875	38.5	7.69
7	15	0	10	1	5.3	31.833	30.5	7.79
8	10	0	15	1	3.1	26.625	19.5	7.76
9	5	0	20	1	2.0	25.042	17.0	8.31
10	0	0	25	1	1.8	22.750	9.0	8.45
11	20	0	5	1	10	8.6	35.417	35.0
12	0	20	5	1	10	1.9	22.042	2.0

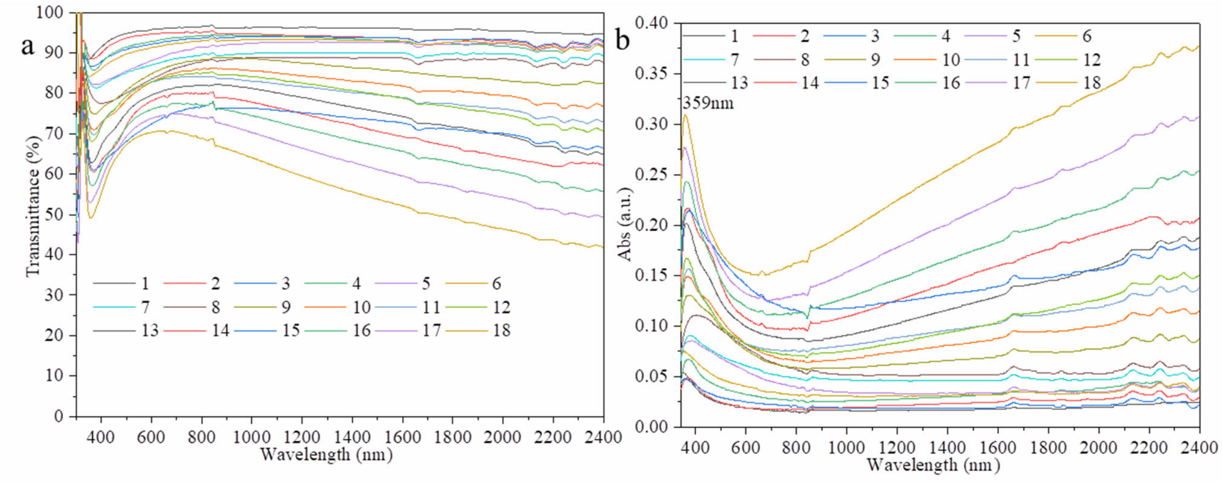

Figure S1. Optical transmittance (a) and (b) absorbance of samples with different printed layers.

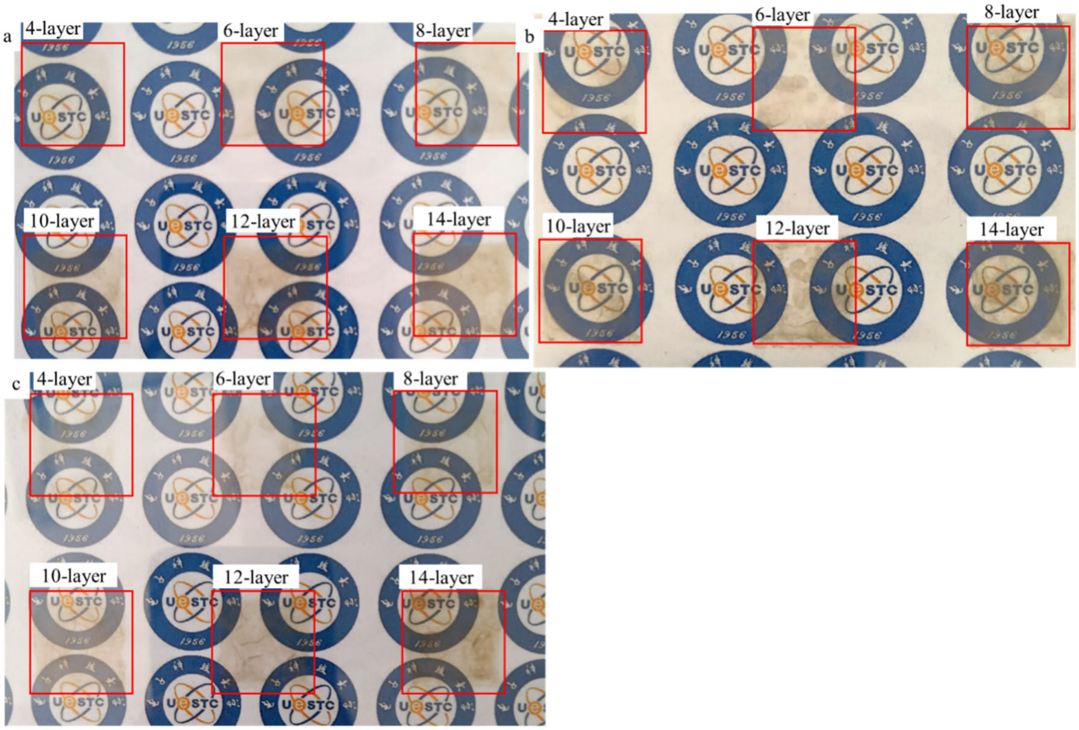

Figure S2 Photographs of printed AgNWs films with different layers and heat treatment at 50 °C (a), 60 °C (b), and 70 °C (c).

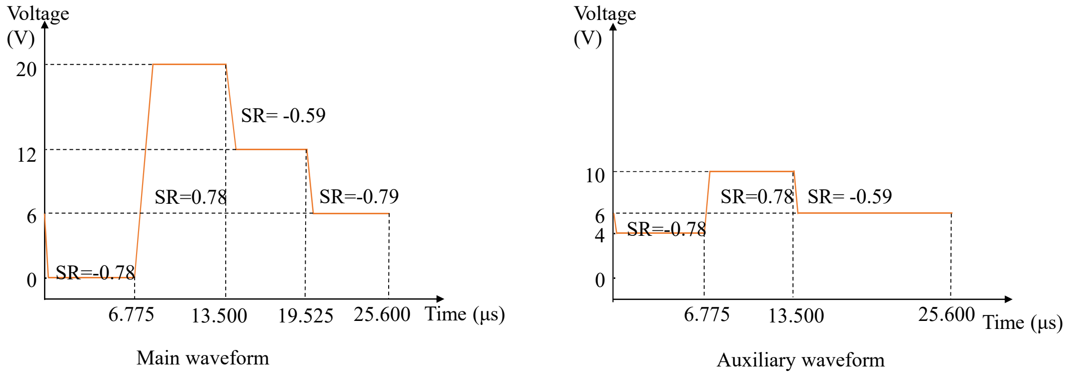

Figure S3 inkjet printing waveform parameters

The main waveform is the parameters of the piezoelectric execution, and the auxiliary waveform connects each of the main waveform. The main waveform is executed first and then the auxiliary waveform is executed. The whole process from the main waveform to the auxiliary waveform performs a piezoelectric inkjet action.
